# Low expression of CIP4 in predicting worse overall survival: A potential biomarker for laryngeal cancer

**DOI:** 10.1371/journal.pone.0253545

**Published:** 2021-09-27

**Authors:** Lucheng Fang, Licai Shi, Wen Wang, Xiu Wu, Tingting Hu, Yideng Huang, Xingwang Rao

**Affiliations:** 1 First Affiliated Hospital of Wenzhou Medical University, Wenzhou, Zhejiang, China; 2 Wenzhou Medical University, Wenzhou, Zhejiang, China; University of Wisconsin, UNITED STATES

## Abstract

Previous reports indicate that Cdc42-interacting protein-4 (CIP4) has previously been reported to plays an important role in the progression of various cancers. However, its correlation with laryngeal cancer (LC) remains unreported. Data from TCGA and GEO databases were used to evaluate the role of CIP4 in LC. Based on GEO and TCGA datasets, we analyzed the differences in CIP4 expression between normal and tumor samples. The Wilcoxon signed-rank test was used to analyze the relationship between clinical features and CIP4. Cox regression and the Kaplan-Meier analyses were used to identify the clinical characteristics associated with the overall survival. Also, the GEPIA database was used to confirm the relationship between CIP4 and overall survival. Lastly, Gene Set Enrichment Analysis (GSEA) was performed based on the TCGA dataset. CIP4 expression in LC was significantly associated with gender and tumor stage (p-values<0.05). Similar to GEPIA validation, Kaplan-Meier survival analysis demonstrated that LC with CIP4-low exhibited a worse prognosis than that with CIP4-high. Univariate analysis revealed that CIP4-high significantly correlated with better overall survival (HR: 0.522, 95% CI: 0.293–0.830, P = 0.026). Besides, multivariate analysis revealed that CIP4 remained independently associated with the overall survival (HR: 0.61, 95% CI: 0.326–0.912, P = 0.012). GSEA showed that the p53, WNT signaling, TGF-β signaling pathways, etc. were enriched in a phenotype high CIP4 expression. In summary, the CIP4 gene is a potential prognostic molecular marker for patients diagnosed with laryngeal cancer. Moreover, the p53, WNT signaling, and TGF-β signaling pathways are potentially associated with CIP4 in LC.

## Introduction

Laryngeal cancer (LC) has a relatively rare occurrence globally. However, it is common with increased age and more prevalent among men than women [1]. In 2018, its global incidence was nearly 180,000, with approximately 100,000 mortalities [2]. Due to the relatively high incidence and mortality of LC, the resulting socio-economic loss and medical burden are enormous. Tobacco and alcohol consumption are the primary significant risk factors for LC. Besides, the function of the human papillomavirus (HPV) is partially implicated in tumorigenesis [3]. According to several previous constructive clinical trials, surgery, radiotherapy, and chemotherapy are the effective treatment approaches of LC based on different tumor stages [4–6]. Moreover, immunotherapy is projected to exert an important influence over the treatment of LC in the future [7]. Whilst acknowledging the notable strides in diagnosis and treatment methods, insignificant change in the 5-year survival rate of LC patients has been noted in recent years [5]. As such, further experiments on the mechanism of occurrence and development of LC are necessary to identify new biomarkers with prognostic value for tumor detection.

Cdc42-interacting protein-4 (CIP4), located on chromosome 19p13.3, is a protein-coding gene. It regulates various cellular processes, including glucose uptake, endocytosis, membrane invagination, among others [8, 9]. Furthermore, previous studies argued that CIP4 is an essential molecule promoting platelet production and cell growth [8, 10–12]. Notably, CIP4 is potentially implicated in cancer pathogenesis. Researchers have extensively reported the roles of CIP4 in various cancers, including nasopharyngeal carcinoma, lung adenocarcinoma, breast tumor, osteosarcoma tumor, etc. [13–16].

Nevertheless, the correlation between CIP4 and LC prognosis of remains unreported. Herein, bioinformatics analysis was performed to detect the difference of CIP4 expression between normal and tumor samples using high throughput RNA-sequencing data from GEO and TCGA databases. Moreover, a survival analysis was performed based on the TCGA profile. This was geared towards evaluating the potential prognostic value and clinical correlation of CIP4 expression in LC. Also, GSEA provided further insights into the biological pathways of the CIP4 regulatory network.

## Methods

### Data acquisition and bioinformatics analysis

Microarray datasets GSE51985 and GSE59102 for analysis were downloaded from the GEO database (https://www.ncbi.nlm.nih.gov/geo/). In total, 10 normal and 10 LC samples were in the GSE51985 dataset (Last update date was Aug 22, 2019; Platform: GPL10558 Illumina HumanHT-12 V4.0 expression beadchip), whereas 13 normal and 29 LC samples were in the GSE59102 dataset (Last update date is Jan 23, 2019; Platform: GPL6480 Agilent-014850 Whole Human Genome Microarray 4x44K G4112F). A total of 12 normal and 111 LC samples were obtained from the TCGA database (The Cancer Genome Atlas Program). Notably, the data category was transcriptome profiling, while the data type was gene expression quantification. Moreover, an experimental strategy was RNA-Seq, while the workflow type was HTSeq—Counts. Clinical characteristic data including gender, age, tumor (T) stage, etc. were simultaneously downloaded. The R software (v.4.0.3) was used to observe a statistical difference (P-value<0.05) in TRIP 10 expression between normal and tumor samples.

### Statistical analysis and GEPIA validation

Based on the median expression values of CIP4, tumor samples were divided into two groups (high CIP4 expression and low CIP4 expression). The survival analysis of CIP4 was performed using the Kaplan-Meier method and log-rank test. The outcome of survival analysis was validated in the GEPIA database, a newly opened interactive web server for cancer and normal gene expression profiling and interactive analyses based on TCGA and the GTEx projects [17]. The tumor type in GEPIA was head and neck squamous cell carcinoma (HNSC), including laryngeal squamous cell carcinoma (group cutoff: median values; 259 normal and 259 tumor samples). The Wilcoxon signed-rank test was used to evaluate statistical differences between clinical pathologic features and CIP4. Univariate Cox regression analysis was used to identify a single factor of clinical characteristics strongly correlated with survival. Besides, multivariate Cox regression analysis was performed to observe the impact of CIP4 expression and other clinical characteristics on survival. All statistical analyses were performed based on R software (v.4.0.3). Furthermore, P-value< 0.05 was considered significant in all statistical analyses.

### Gene Set Enrichment Analysis (GSEA)

Gene Set Enrichment Analysis (GSEA) software version 3.0 (http://www.gsea-msigdb.org/gsea/index.jsp) was used for functional enrichment analysis. First, genes were ranked in GSEA based on the correlation between their expression and CIP4 expression. Subsequently, GSEA was conducted to identify significant signaling pathways between low and high CIP4 expression datasets. The annotated gene set files (c2.cp.kegg.v7.0.symbols.gmt and h.all.c2.v7.2.symbols.gmt) served as references. For each analysis, gene set permutations were performed 1,000 times. The phenotype label was CIP4 expression level. Besides, signaling pathways of gene sets with the nominal (NOM) P-val <0.05 and the false discovery rate (FDR) q-val <0.25 were considered significant.

## Results

### Comparison between CIP4 expression and patient clinical characteristics

**Fig 1:**
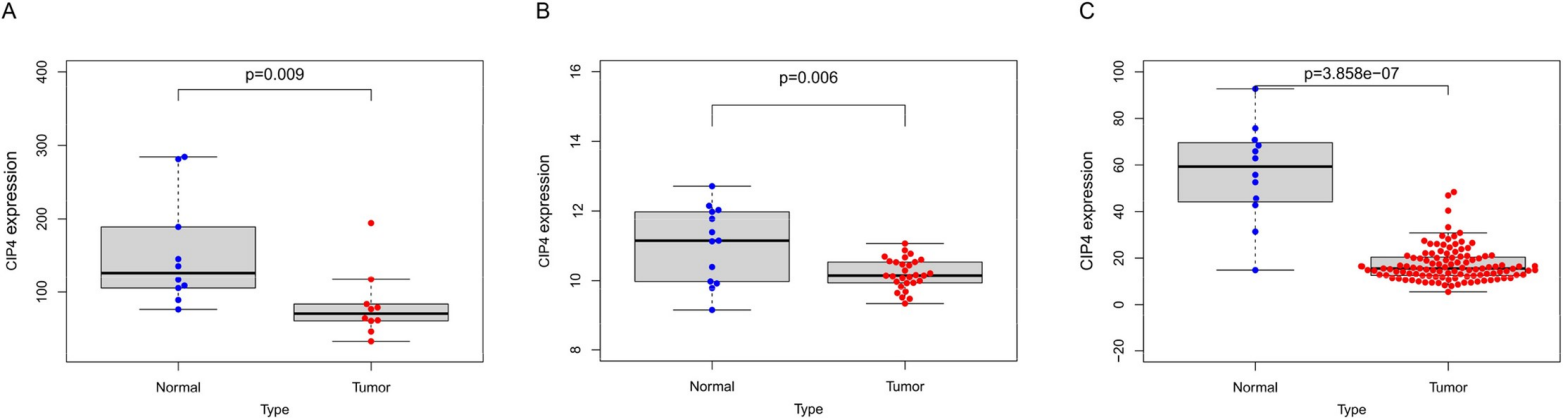
The expression levels of CIP4 between tumor and non-tumor samples in LC patients. (A) GSE51985; (B) GSE59102; (C) TCGA database.

Three datasets were adopted to investigate the difference in CIP4 expression levels between LC and normal tissues. As shown in Fig 1A, CIP4 expression in tumor tissue samples (n = 10) was significantly lower than in normal tissue samples (n = 10) in GSE51985 (P = 0.009). In GSE59102, significantly lower expression of CIP4 was noted in 29 LC patients than in 13 normal samples (P = 0.006, Fig 1B). Moreover, a similar trend was observed between tumor (n = 111) and normal samples (n = 12) in the TCGA dataset (P<0.001, Fig 1C). Clinical characteristics of 111 LC patients from TCGA are displayed in Table 1.

**Table 1:** TCGA laryngeal cancer patient characteristics.

Clinical characteristics		Total(111)	%
Age(year)	≤60	48	43.2
	>60	63	56.8
Gender	Female	22	19.8
	Male	89	80.2
AJCC stage	Stage I	3	2.7
	Stage II	11	9.9
	Stage III	26	23.4
	Stage IV(A.B.C)	67	60.4
	NA	4	3.6
Tumor stage	T1	3	2.7
	T2	17	15.3
	T3	35	31.5
	T4	52	46.8
	TX	3	2.7
	NA	1	0.9
Lymph node status	N0	55	49.5
	N1	18	16.2
	N2	29	26.1
	N3	3	2.7
	NX	5	4.5
	NA	1	0.9
Metastasis	M0	104	93.7
	M1	2	1.8
	MX	3	2.7
	NA	2	1.8
Race	No White	21	18.9
	White	86	77.5
	NA	4	3.6
Alcohol history	Yes	70	63.1
	No	39	35.1
	NA	2	1.8
Smoking history	Yes	74	66.7
	No	37	33.3

### Survival outcomes and cox analysis

**Fig 2:**
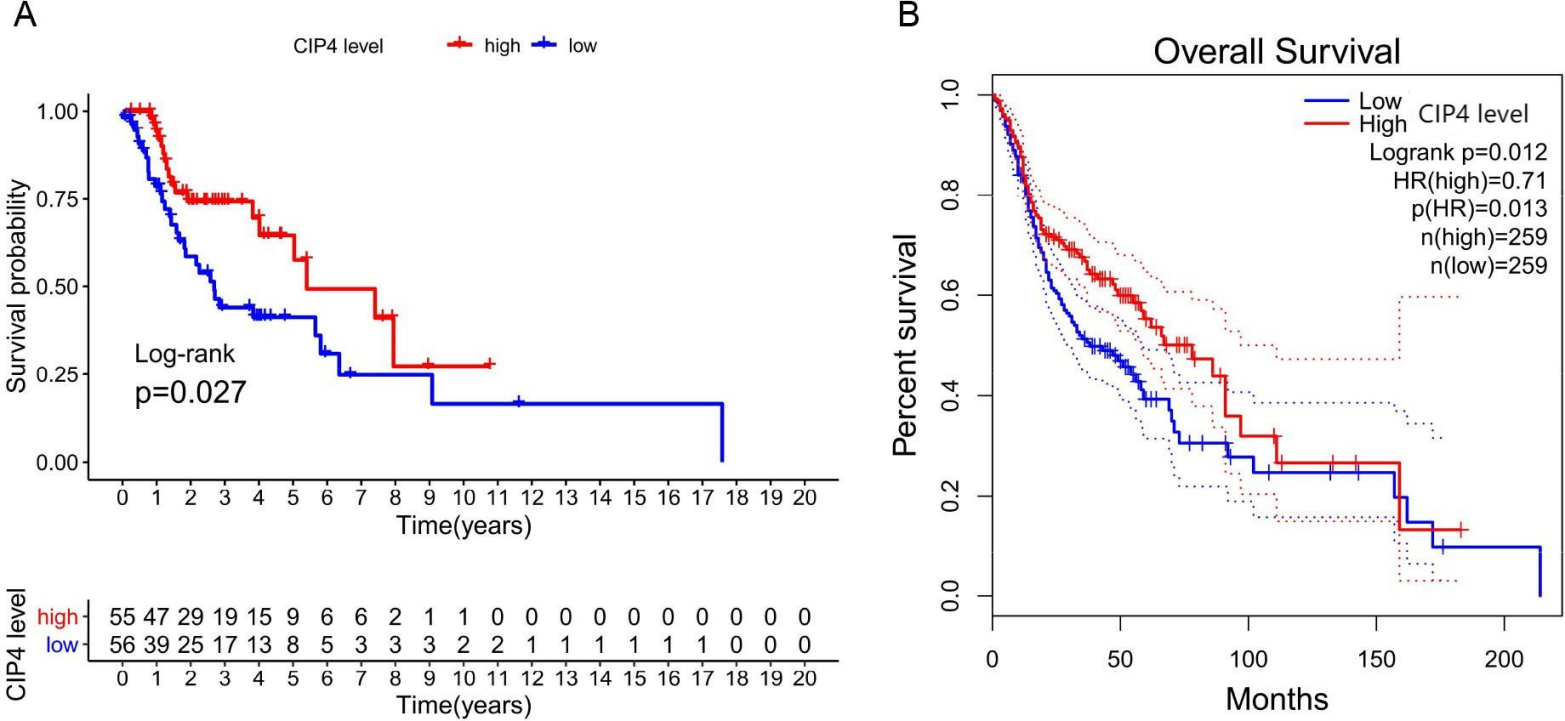
(A) Impact of CIP4 expression on overall survival in LC patients in TCGA cohort; (B) Impact of CIP4 expression on overall survival in GEPIA database.

As shown in Fig 2A, Kaplan-Meier survival analysis indicated that tumor tissue with low expression of CIP4 was considerably linked to worse overall survival (P = 0.026). Similarly, the survival analysis in the GEPIA database showed that low CIP4 expression in tumor tissues had worse overall survival than CIP4-high expression (P = 0.012, Fig 2B). According to univariate analysis, five factors including CIP4 expression levels (HR:0.522, 95% CI:0.293–0.830, P = 0.0027), gender (HR:0.297 95% CI:0.151–0.586, P<0.001), tumor stage (HR:1.451, 95% CI:1.363–1.656, P = 0.044), lymph node status (HR:1.894, 95% CI:1.060–3.383, P = 0.031) and metastasis (HR:4.86, 95% CI:1.670–14.146, P = 0.004) exerted significant influence over survival. However, based on multivariate analysis, CIP4 expression (HR: 0.61, 95% CI: 0.326–0.912, P = 0.012) remained associated with overall survival, along with gender (HR: 0.375 95% CI: 0.179–0.784 P = 0.009), tumor stage (HR: 1.547, 95% CI: 1.497–4.771, P = 0.045) and metastasis (HR: 2.031, 95% CI: 1.059–3.897, P = 0.033). Comprehensive information of Cox analysis is shown in Table 2.

**Table 2:** Univariate and multivariable cox regression analysis on OS.

	Univariate Cox					Multivariate Cox	
Variables	HR	95% CI	P-value		HR	95% CI	P-value
CIP4 (high vs. low)	0.522	0.293–0.830	0.027		0.61	0.326–0.912	0.012
Age(>60 vs.≤60)	0.806	0.454–1.429	0.46		0.738	0.399–1.366	0.334
Gender(male vs.female)	0.297	0.151–0.586	<0.001		0.375	0.179–0.784	0.009
AJCC stage(III-IV vs.I-II)	0.782	0.366–1.672	0.057		0.568	0.174–1.861	0.351
Tumor stage (3–4 vs.1-2)	1.451	1.363–1.656	0.044		1.547	1.497–4.771	0.045
Lymph node status(2–3 vs.0-1)	1.894	1.060–3.383	0.031		2.823	0.887–8.990	0.079
Metastasis(1 vs.0)	4.86	1.670–14.146	0.004		2.031	1.059–3.897	0.033
Race(White vs.No white)	1.421	0.748–2.699	0.284		1.373	0.689–2.734	0.367
Alcohol history(No vs.Yes)	1.497	0.846–2.650	0.166		1.528	0.794–2.940	0.204
Smokinghistory(No vs.Yes)	1.061	0.588–1.912	0.166		1.047	0.566–1.938	0.884

Abbreviations: CI, confidence interval; HR, hazard ratio; OS, overall survival.

### Relationship between CIP4 expression and clinicopathologic features in LC patients

**Fig 3:**
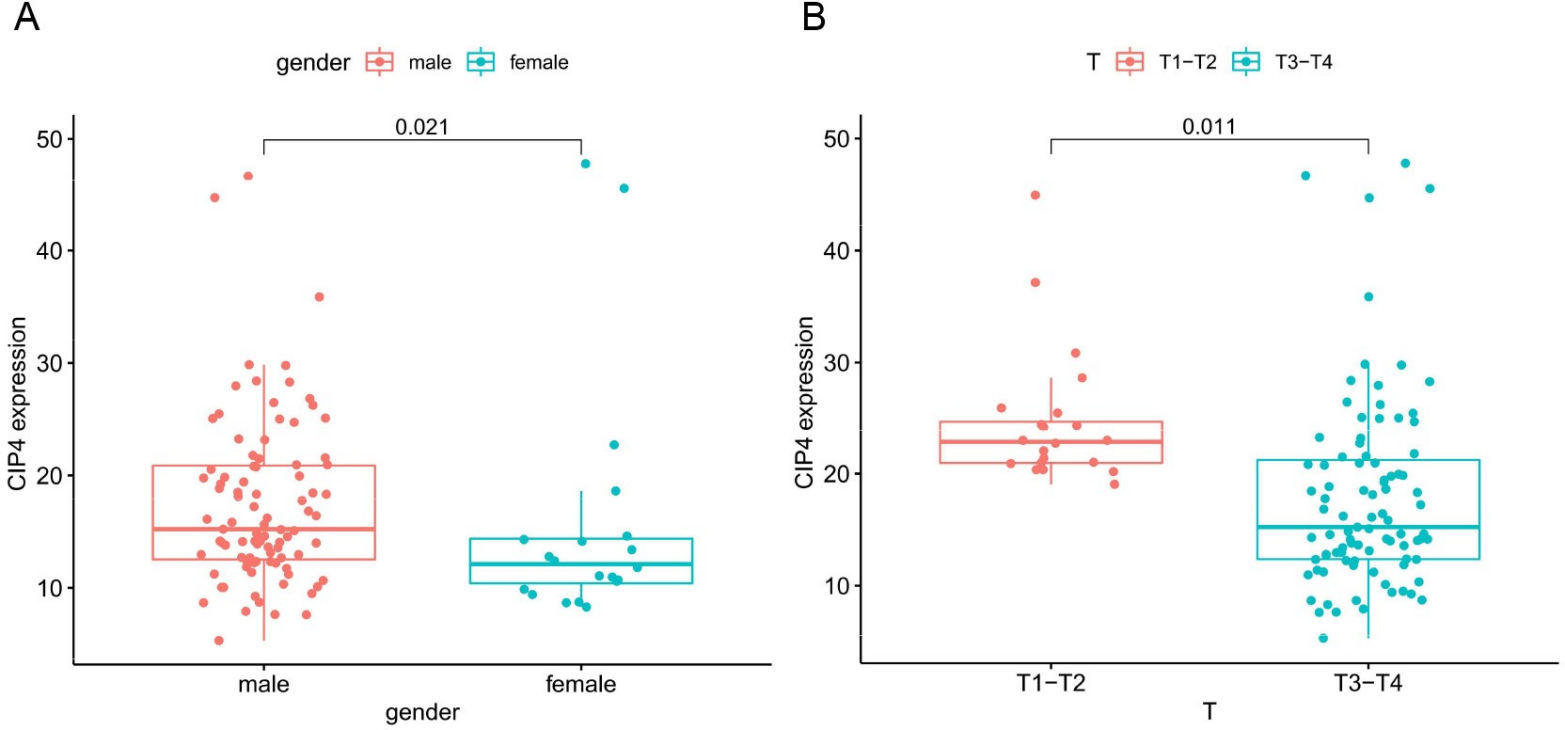
Relationship between CIP4 expression and clinical characteristics. (A) Gender; (B) Tumor stage.

As shown in S1–S7 Figs, age, AJCC stage, lymph node status, metastasis, race, alcohol history, and smoking history were not significantly different in CIP4 expression (P> 0.05). On the other hand, the expression level of CIP4 was significantly related to gender (Fig 3A) and tumor stage (Fig 3B). Upregulated CIP4 expression significantly correlated with low tumor-stage (P = 0.011) and male (P = 0.021).

### CIP4-related potential signaling pathways based on GSEA

**Fig 4:**
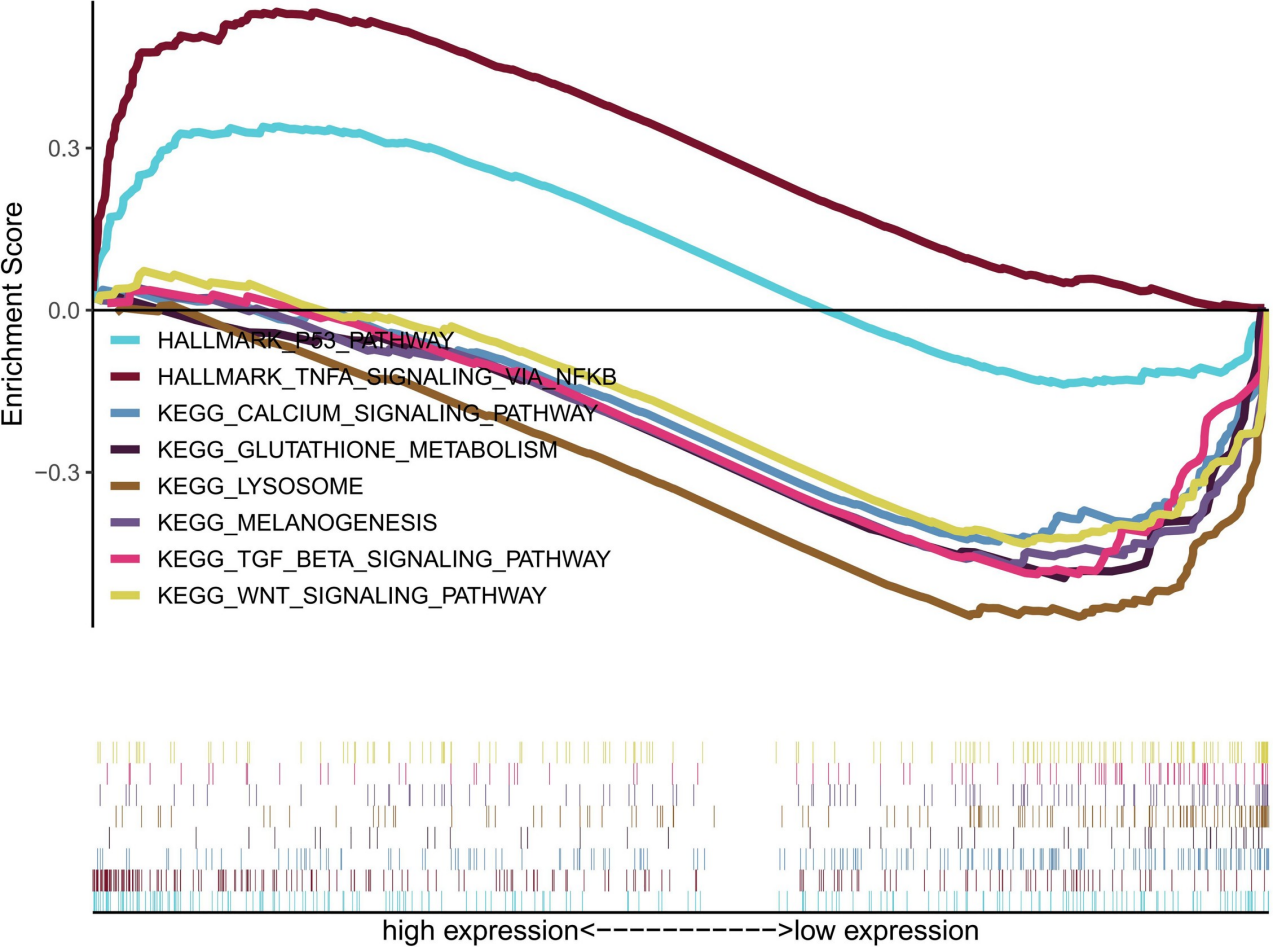
Enrichment plots from Gene Set Enrichment Analysis (GSEA).

GSEA was conducted to evaluate the potential biological mechanism related to CIP4 expression. The most significantly enriched signaling pathways were identified following their normalized enrichment score (NES). As delineated in Fig 4, the GSEA revealed that CIP4 expression was linked to “TNF-α signaling via NF-kB”, “p53 pathway”, “glutathione metabolism”, “calcium signaling pathway”, “WNT signaling pathway”, “TGF-β signaling pathway”, “melanogenesis” and “Lysosome”. The detailed information of signaling pathways is shown in Table 3.

**Table 3:** Gene set enrichment analysis.

Gene set name	NES	NOM p-val	FDR q-val
HALLMARK_TNFA_SIGNALING_VIA_NFKB	1.74	0.024	0.046
HALLMARK_P53_PATHWAY	1.437	0.017	0.201
KEGG_GLUTATHIONE_METABOLISM	-1.545	0.039	0.192
KEGG_CALCIUM_SIGNALING_PATHWAY	-1.575	0.006	0.186
KEGG_WNT_SIGNALING_PATHWAY	-1.596	0.010	0.173
KEGG_TGF_BETA_SIGNALING_PATHWAY	-1.651	0.020	0.150
KEGG_MELANOGENESIS	-1.698	0.010	0.141
KEGG_LYSOSOME	-1.88	0.004	0.129

NES: normalized enrichment score; NOM: nominal; FDR: false discovery rate. Gene sets with NOM p-val <0.05 and FDR q-val <0.25 are considered as significant.

## Discussion

Laryngeal carcinoma (LC) is the second most prevalent head and neck cancer with rising mortality rates in the United States [6]. Identifying novel biomarkers is necessary for its diagnosis, treatment, and prognostic assessment. To our knowledge, the CIP4 function and its potential prognostic impact on LC remain unreported. Cdc42-interacting protein-4 (CIP4), also known as a thyroid hormone receptor interactor 10 (TRIP10) belongs to the F-BAR protein subfamily. Structurally, it contains F-BAR, HR1, and SH3 domains [18]. CIP4 is implicated in the mechanism of endocytosis recruiting Wiskott-Aldrich syndrome protein (WASP) and GTPase dynamin via the SH3 domain [19]. Moreover, reports indicate that the F-BAR domain of CIP4 is linked to receptor trafficking and cell cycle progress [20]. Besides, previous research revealed that CIP4 inhibits neurite formation dependent on the F-BAR and SH3 domains [21].

Notably, CIP4 has an important role in diseases and developmental disorders. In *drosophila* embryogenesis, CIP4 overexpression prevents actin from nucleation, thereby causing developmental disorder [22]. Besides, the relationship between CIP4 overexpression and the death of striatal neurons is related to the pathogenesis of Huntington’s disease [23]. Rusconi and his colleagues reported that myocyte hypertrophy was inhibited due to CIP4 knockdown in a rat; a recombinant CIP4 rescued this inhibition [10]. In recent years, the expression and functions of CIP4 in cancers have elicited increasing attention and are actively investigated [11, 13–16]. Several studies indicate that CIP4 regulates the invasiveness and metastasis of cancer cells. Strong CIP4 expression demonstrated a positive correlation with metastasis of nasopharyngeal carcinoma by activating EGFR signaling [13]. Additionally, one study discovered that CIP4 overexpression, relevant to poor prognosis, promoted lung adenocarcinoma metastasis [14]. Based on in vitro and in vivo experiments involving CIP4 phosphorylation by PKA during the acquisition of a metastatic phenotype in cancer cells, Tonucci et al. [11], found a signaling pathway. Besides, CIP4 promotes endocytosis of transmembrane type I matrix metalloprotease (MT1-MMP), potentially suppressing breast tumor cell invasion [15]. The above findings provide a constructive basis for further investigations on the CIP4 mechanisms in laryngeal carcinoma.

This paper asserts that the relatively upregulated CIP4 expression in LC correlates with the tumor stage and predicted poor prognosis. Consequently, CIP4 exerts an important influence over LC in tumorigenesis and metastasis. To further investigate CIP4 roles in LC, GSEA was applied using TCGA data. As a consequence, TNF-α signaling via NF-kB, P53 pathway, glutathione metabolism, calcium signaling pathway, WNT signaling pathway, TGF-β signaling pathway, melanogenesis, and Lysosome were differentially enriched in high CIP4 expression phenotype.

Limited information is available on the molecular mechanism of the aforementioned signaling pathways in laryngeal squamous cell cancer. Previous studies revealed that p53 overexpression was remarkably associated with laryngeal carcinoma as well as head and neck squamous cell carcinoma in the immunohistochemical analysis [24, 25]. Based on the antitumor effect mediated by p53, a recent study also reported that Lupeol regulates neoplastic growth and apoptosis in laryngeal cancer [26]. A study by Fountzilas’s group reported a significant relationship between downregulation of the WNT signaling pathway and prognosis of LC patients [27, 28]. According to Zhang’s report, CIP4 silencing alleviates streptozotocin-induced pulmonary fibrosis in mice by suppressing the Wnt pathway [29]. Moreover, the TGF-β signaling pathway enhances cell proliferation and the survival rate of fibroblasts [30]. Chen and his colleagues identified that upregulated miR-141 inhibits the TGF-β signaling pathway, thereby decreasing epithelial-mesenchymal transition and metastasis of LC [31]. Similarly, the upregulation of CIP4 accelerated the process of epithelial-mesenchymal transition induced by transforming growth factor-β (TGF-β) [32]. Furthermore, as proteins with SH3 domain, CIP4 potentially played a vital role in the subcellular distribution and lysosomal association of Fas Ligand (FasL) [33]. In summary, we believe that the aforementioned signaling pathways associated with CIP4 exert a strong impact on LC.

This study has compelling limitations. First, the sample size was relatively inadequate. No experimental study was conducted to explore the potential carcinogenic mechanism of CIP4 in the development of LC. Additional studies are essential to shed light on the precise functional mechanisms of CIP4 in LC. Besides, information acquired from all databases was limited, therefore improvement of the databases will lead to varied and credible outcomes.

## Conclusion

In conclusion, this work postulated that CIP4 is a potential prognostic factor in LC patients. The CIP4 expression level was downregulated with tumor progression. Besides, the p53, WNT signaling, and TGF-β signaling pathways are potentially associated with CIP4 in LC. However, it is necessary to conduct further experimental validation, including molecular mechanisms and deeper genomic research to verify the biological impact of CIP4.

## Supporting information

S1 Fig Correlation between CIP4 expression and age. (JPG)

S2 Fig Correlation between CIP4 expression and AJCC stage. (JPG)

S3 Fig Correlation between CIP4 expression and lymph node status. (JPG)

S4 Fig Correlation between CIP4 expression and metastasis. (JPG)

S5 Fig Correlation between CIP4 expression and race. (JPG)

S6 Fig Correlation between CIP4 expression and alcohol history. (JPG)

S7 Fig Correlation between CIP4 expression and smoking history. (JPG)

## References

[1] Ferster APOC, Schubart J, Kim Y, Goldenberg D. Association Between Laryngeal Cancer and Asbestos Exposure: A Systematic Review. JAMA Otolaryngol Head Neck Surg. 2017; 143:409–416. doi: 10.1001/jamaoto.2016.3421 27918783

[2] Bray F, Ferlay J, Soerjomataram I, Siegel R, Torre L, Jemal A. Global cancer statistics 2018: GLOBOCAN estimates of incidence and mortality worldwide for 36 cancers in 185 countries. CA Cancer J Clin. 2018; 68:394–424. doi: 10.3322/caac.21492 30207593

[3] Steuer CE, El-Deiry M, Parks JR, Higgins KA, Saba NF. An update on larynx cancer. CA Cancer J Clin. 2017; 67:31–50. doi: 10.3322/caac.21386 27898173

[4] Warner L, Chudasama J, Kelly CG, Loughran S, McKenzie K, Wight R, et al. Radiotherapy versus open surgery versus endolaryngeal surgery (with or without laser) for early laryngeal squamous cell cancer. Cochrane Database Syst Rev. 2014:CD002027. doi: 10.1002/14651858.CD002027.pub2 25503538 PMC6599864

[5] Obid R, Redlich M, Tomeh C. The Treatment of Laryngeal Cancer. Oral Maxillofac Surg Clin North Am. 2019; 31:1–11. doi: 10.1016/j.coms.2018.09.001 30449522

[6] Baird BJ, Sung CK, Beadle BM, Divi V. Treatment of early-stage laryngeal cancer: A comparison of treatment options. Oral Oncol. 2018; 87. doi: 10.1016/j.oraloncology.2018.09.012 30527248

[7] Carlisle JW, Steuer CE, Owonikoko TK, Saba NF. An update on the immune landscape in lung and head and neck cancers. CA Cancer J Clin. 2020; 70:505–517. doi: 10.3322/caac.21630 32841388

[8] Hsu C, Leu Y, Tseng M, Lee K, Kuo T, Yen J, et al. Functional characterization of Trip10 in cancer cell growth and survival. J Biomed Sci. 2011; 18:12. doi: 10.1186/1423-0127-18-12 21299869 PMC3044094

[9] Hartig SM, Ishikura S, Hicklen RS, Feng Y, Blanchard EG, Voelker KA, et al. The F-BAR protein CIP4 promotes GLUT4 endocytosis through bidirectional interactions with N-WASp and Dynamin-2. J Cell Sci. 2009; 122:2283–2291. doi: 10.1242/jcs.041343 19509061 PMC2723146

[10] Rusconi F, Thakur H, Li J, Kapiloff M. CIP4 is required for the hypertrophic growth of neonatal cardiac myocytes. J Biomed Sci. 2013; 20:56. doi: 10.1186/1423-0127-20-56 23915320 PMC3750294

[11] Tonucci F, Almada E, Borini-Etichetti C, Pariani A, Hidalgo F, Rico M, et al. Identification of a CIP4 PKA phosphorylation site involved in the regulation of cancer cell invasiveness and metastasis. Cancer Lett. 2019; 461:65–77. doi: 10.1016/j.canlet.2019.07.006 31319138

[12] Chen Y, Aardema J, Kale S, Whichard ZL, Awomolo A, Blanchard E, et al. Loss of the F-BAR protein CIP4 reduces platelet production by impairing membrane-cytoskeleton remodeling. Blood. 2013; 122:1695–1706. doi: 10.1182/blood-2013-03-484550 23881916 PMC3765055

[13] Meng D, Xie P, Peng L, Sun R, Luo D, Chen Q, et al. CDC42-interacting protein 4 promotes metastasis of nasopharyngeal carcinoma by mediating invadopodia formation and activating EGFR signaling. J Exp Clin Cancer Res. 2017; 36:21. doi: 10.1186/s13046-016-0483-z 28129778 PMC5273811

[14] Truesdell P, Ahn J, Chander H, Meens J, Watt K, Yang X, et al. CIP4 promotes lung adenocarcinoma metastasis and is associated with poor prognosis. Oncogene. 2015; 34:3527–3535. doi: 10.1038/onc.2014.280 25174397 PMC4978543

[15] Hu J, Mukhopadhyay A, Truesdell P, Chander H, Mukhopadhyay U, Mak A, et al. Cdc42-interacting protein 4 is a Src substrate that regulates invadopodia and invasiveness of breast tumors by promoting MT1-MMP endocytosis. J Cell Sci. 2011; 124:1739–1751. doi: 10.1242/jcs.078014 21525036

[16] Koshkina NV, Yang G, Kleinerman ES. Inhibition of Cdc42-interacting protein 4 (CIP4) impairs osteosarcoma tumor progression. Curr Cancer Drug Targets. 2013; 13:48–56. 22920438

[17] Tang Z, Li C, Kang B, Gao G, Li C, Zhang Z. GEPIA: a web server for cancer and normal gene expression profiling and interactive analyses. Nucleic Acids Res. 2017; 45. doi: 10.1093/nar/gkx247 28407145 PMC5570223

[18] Liu S, Xiong X, Zhao X, Yang X, Wang H. F-BAR family proteins, emerging regulators for cell membrane dynamic changes-from structure to human diseases. J Hematol Oncol. 2015; 8:47. doi: 10.1186/s13045-015-0144-2 25956236 PMC4437251

[19] Feng Y, Hartig S, Bechill J, Blanchard E, Caudell E, Corey S. The Cdc42-interacting protein-4 (CIP4) gene knock-out mouse reveals delayed and decreased endocytosis. J Biol Chem. 2010; 285:4348–4354. doi: 10.1074/jbc.M109.041038 19920150 PMC2836039

[20] Hu J, Troglio F, Mukhopadhyay A, Everingham S, Kwok E, Scita G, et al. F-BAR-containing adaptor CIP4 localizes to early endosomes and regulates Epidermal Growth Factor Receptor trafficking and downregulation. Cell Signal. 2009; 21:1686–1697. doi: 10.1016/j.cellsig.2009.07.007 19632321

[21] Saengsawang W, Mitok K, Viesselmann C, Pietila L, Lumbard D, Corey S, et al. The F-BAR protein CIP4 inhibits neurite formation by producing lamellipodial protrusions. Curr Biol. 2012; 22:494–501. doi: 10.1016/j.cub.2012.01.038 22361215 PMC3311763

[22] Yan S, Lv Z, Winterhoff M, Wenzl C, Zobel T, Faix J, et al. The F-BAR protein Cip4/Toca-1 antagonizes the formin Diaphanous in membrane stabilization and compartmentalization. J Cell Sci. 2013; 126:1796–1805. doi: 10.1242/jcs.118422 23424199 PMC3706074

[23] Holbert S, Dedeoglu A, Humbert S, Saudou F, Ferrante R, Néri C. Cdc42-interacting protein 4 binds to huntingtin: neuropathologic and biological evidence for a role in Huntington’s disease. Proc Natl Acad Sci U S A. 2003; 100:2712–2717. doi: 10.1073/pnas.0437967100 12604778 PMC151406

[24] Nadal A, Cardesa A. Molecular biology of laryngeal squamous cell carcinoma. Virchows Arch. 2003; 442:1–7. doi: 10.1007/s00428-002-0726-6 12536308

[25] Karpathiou G, Monaya A, Forest F, Froudarakis M, Casteillo F, Marc Dumollard J, et al. p16 and p53 expression status in head and neck squamous cell carcinoma: a correlation with histological, histoprognostic and clinical parameters. Pathology. 2016; 48:341–348. doi: 10.1016/j.pathol.2016.01.005 27113547

[26] Bhattacharyya S, Sekar V, Majumder B, Mehrotra DG, Banerjee S, Bhowmick AK, et al. CDKN2A-p53 mediated antitumor effect of Lupeol in head and neck cancer. Cell Oncol (Dordr). 2017; 40:145–155. doi: 10.1007/s13402-016-0311-7 28039610 PMC13001573

[27] Fountzilas E, Kotoula V, Angouridakis N, Karasmanis I, Wirtz RM, Eleftheraki AG, et al. Identification and validation of a multigene predictor of recurrence in primary laryngeal cancer. PLoS One. 2013; 8:e70429. doi: 10.1371/journal.pone.0070429 23950933 PMC3739775

[28] Psyrri A, Kotoula V, Fountzilas E, Alexopoulou Z, Bobos M, Televantou D, et al. Prognostic significance of the Wnt pathway in squamous cell laryngeal cancer. Oral Oncol. 2014; 50:298–305. doi: 10.1016/j.oraloncology.2014.01.005 24461629

[29] Zhang X, Liu Y, Shao R, Li W. Cdc42-interacting protein 4 silencing relieves pulmonary fibrosis in STZ-induced diabetic mice via the Wnt/GSK-3β/β-catenin pathway. Exp Cell Res. 2017; 359:284–290. doi: 10.1016/j.yexcr.2017.07.018 28720386

[30] Achavanuntakul K, Charoenkwan K. Factors affecting operative blood loss from open radical hysterectomy and pelvic lymphadenectomy for early-stage cervical cancer. Arch Gynecol Obstet. 2012; 286:1001–1005. doi: 10.1007/s00404-012-2387-2 22622853

[31] Chen L, Sun D-Z, Fu Y-G, Yang P-Z, Lv H-Q, Gao Y, et al. Upregulation of microRNA-141 suppresses epithelial-mesenchymal transition and lymph node metastasis in laryngeal cancer through HOXC6-dependent TGF-β signaling pathway. Cell Signal. 2020; 66:109444. doi: 10.1016/j.cellsig.2019.109444 31629025

[32] Zhu Y, Wang Y, Bai S, Zha F, Feng G, Gao C, et al. Suppression of CIP4/Par6 attenuates TGF-β1-induced epithelial-mesenchymal transition in NRK-52E cells. Int J Mol Med. 2017; 40:1165–1171. doi: 10.3892/ijmm.2017.3100 28848997 PMC5593455

[33] Qian J, Chen W, Lettau M, Podda G, Zörnig M, Kabelitz D, et al. Regulation of FasL expression: a SH3 domain containing protein family involved in the lysosomal association of FasL. Cell Signal. 2006; 18:1327–1337. doi: 10.1016/j.cellsig.2005.10.015 16318909

