# Supplementary figures and images for: Low expression of CIP4 in predicting worse overall survival: A potential biomarker for laryngeal cancer
Source: PLoS One. 2021 Sep 27;16(9):e0253545. doi: 10.1371/journal.pone.0253545 (PMC8475988; doi:10.1371/journal.pone.0253545)

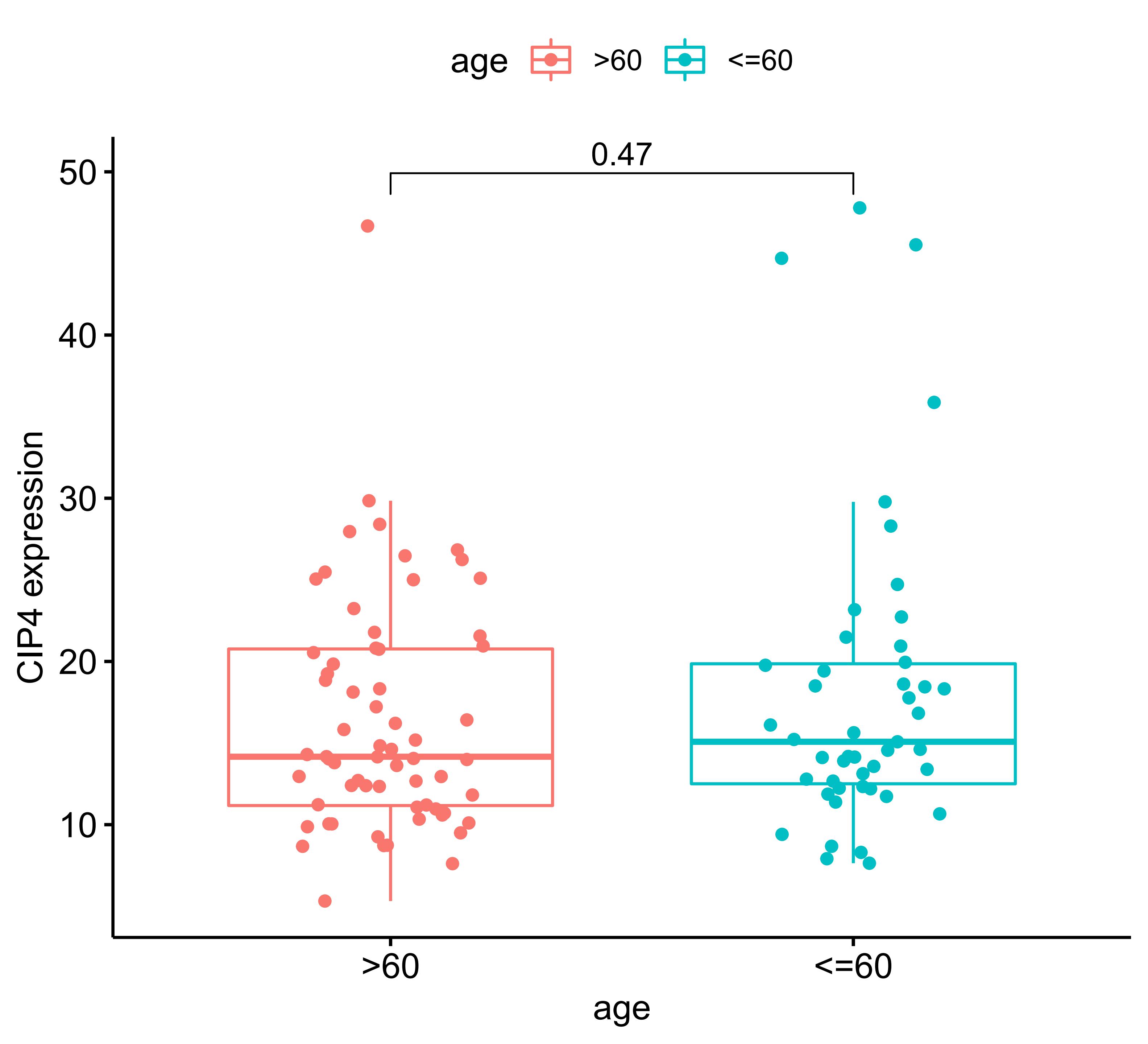

Supplement: S1 Fig — (JPG) [file pone.0253545.s001.jpg]

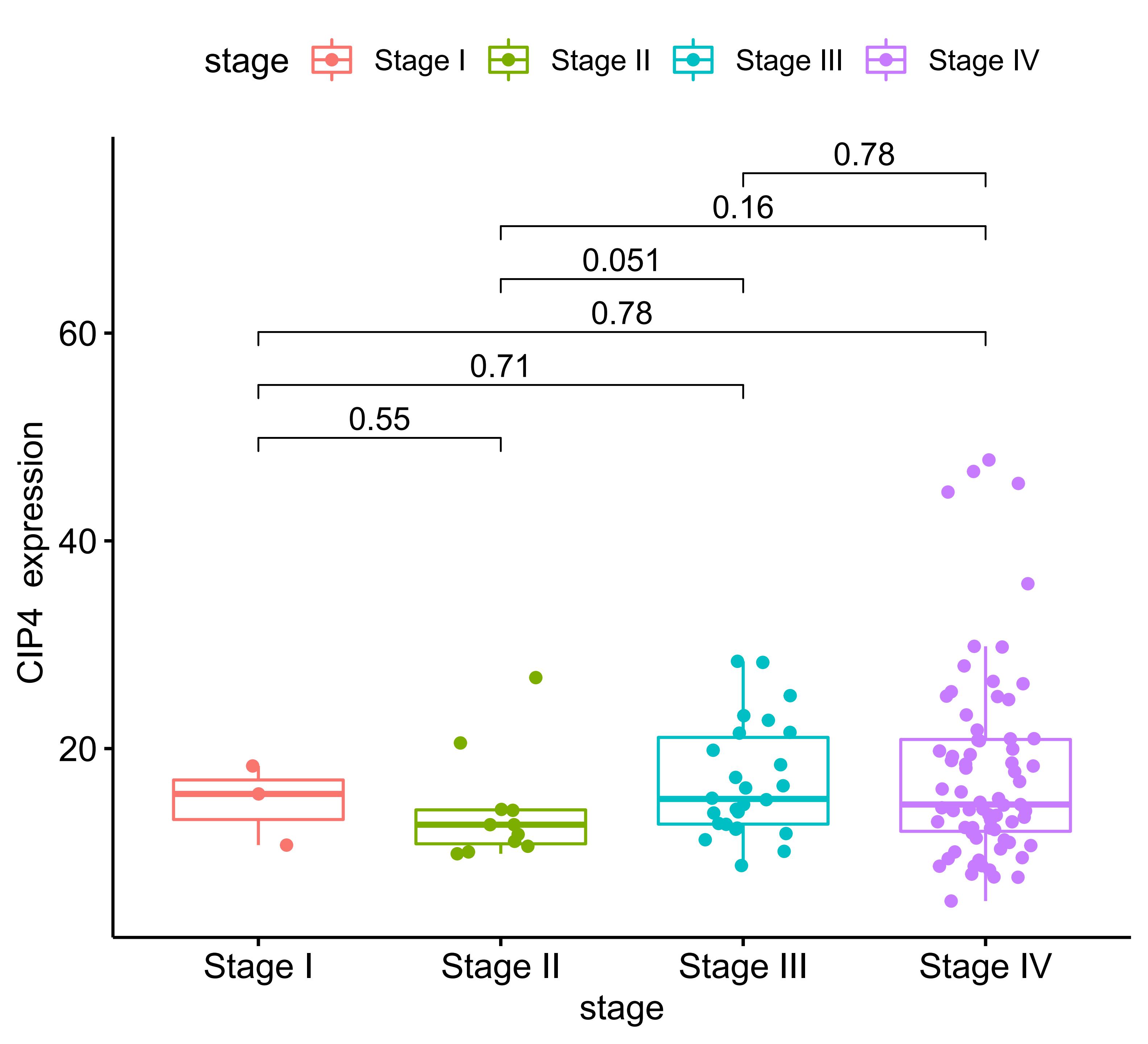

Supplement: S2 Fig — (JPG) [file pone.0253545.s002.jpg]

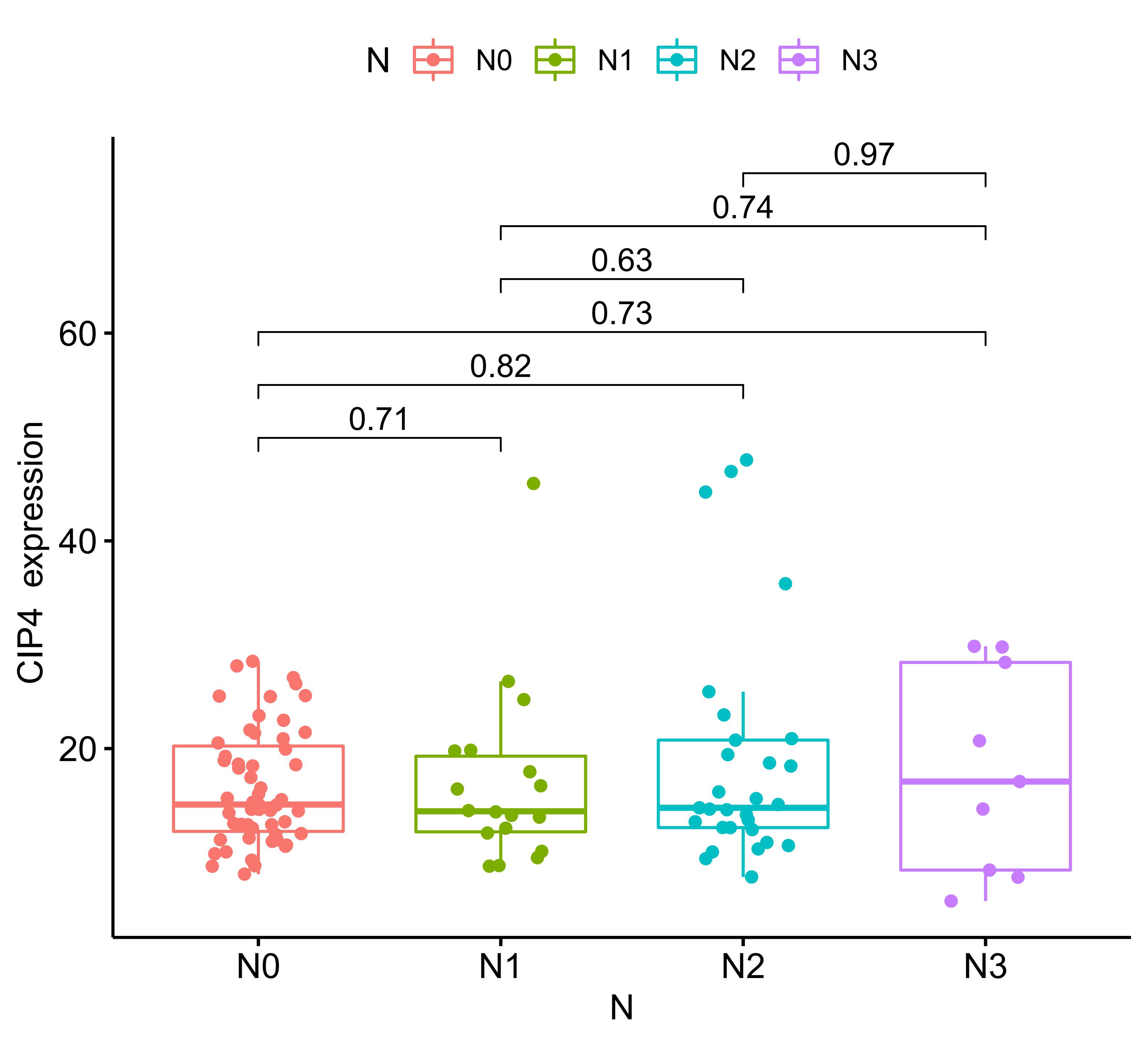

Supplement: S3 Fig — (JPG) [file pone.0253545.s003.jpg]

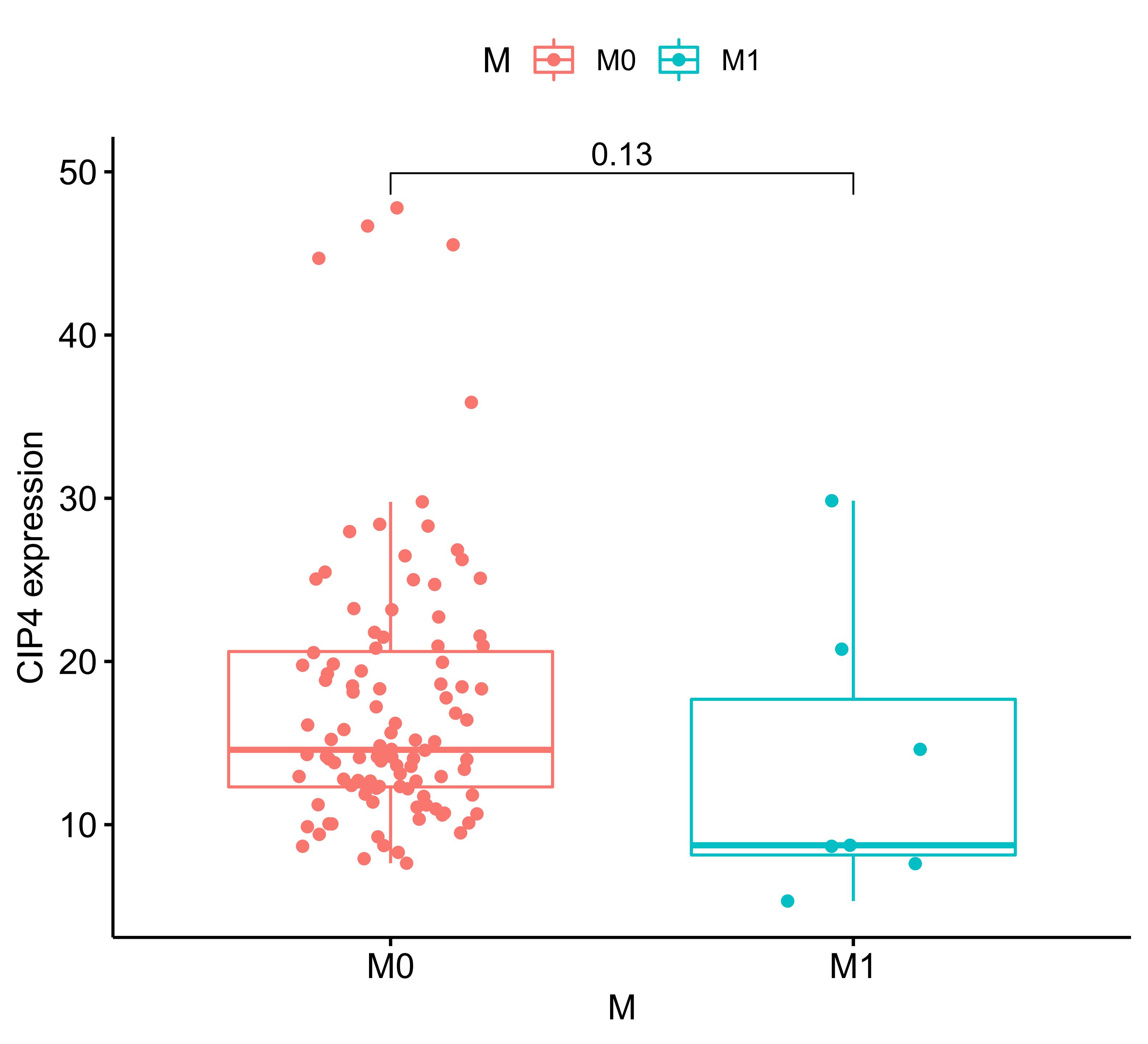

Supplement: S4 Fig — (JPG) [file pone.0253545.s004.jpg]

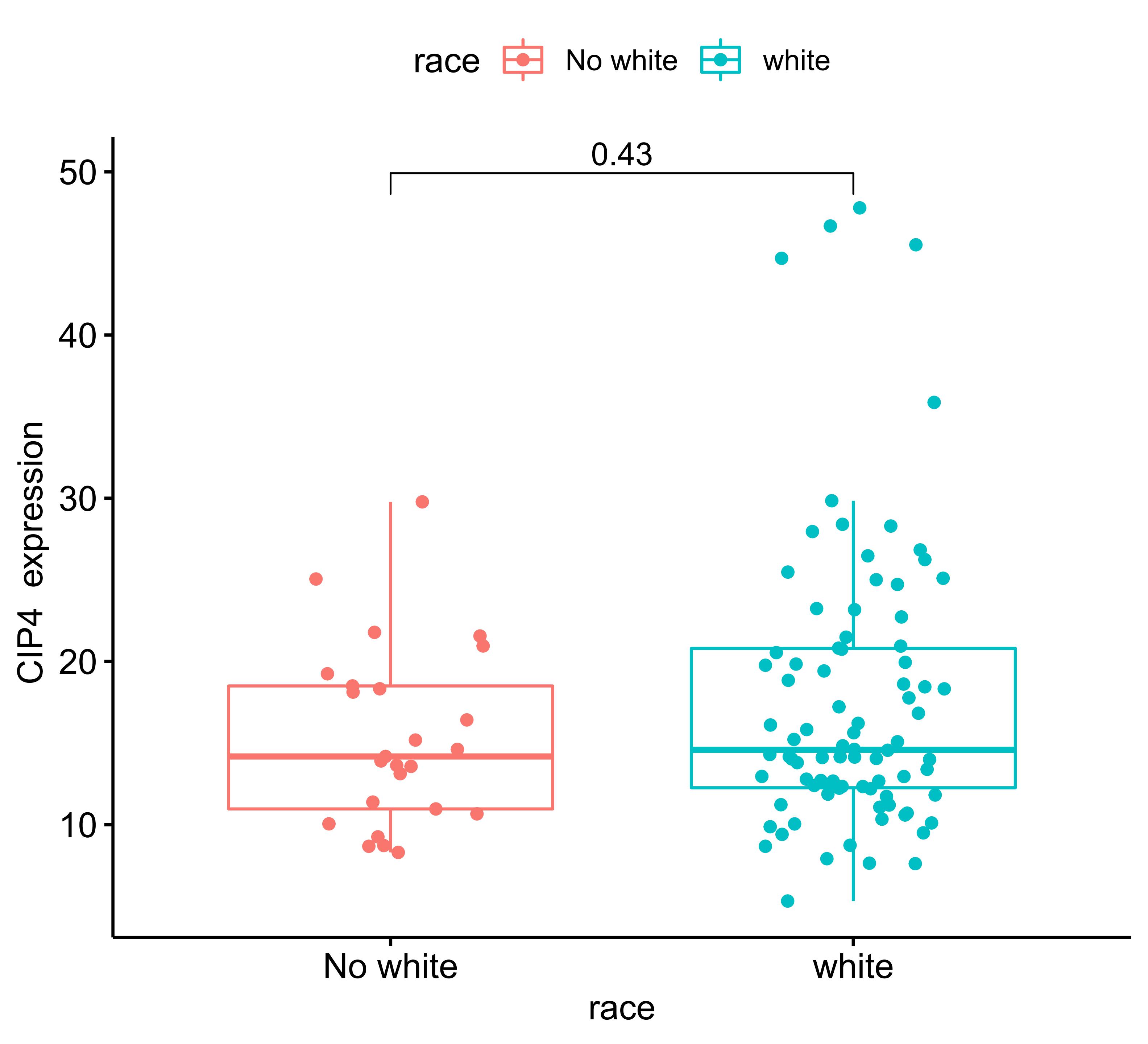

Supplement: S5 Fig — (JPG) [file pone.0253545.s005.jpg]

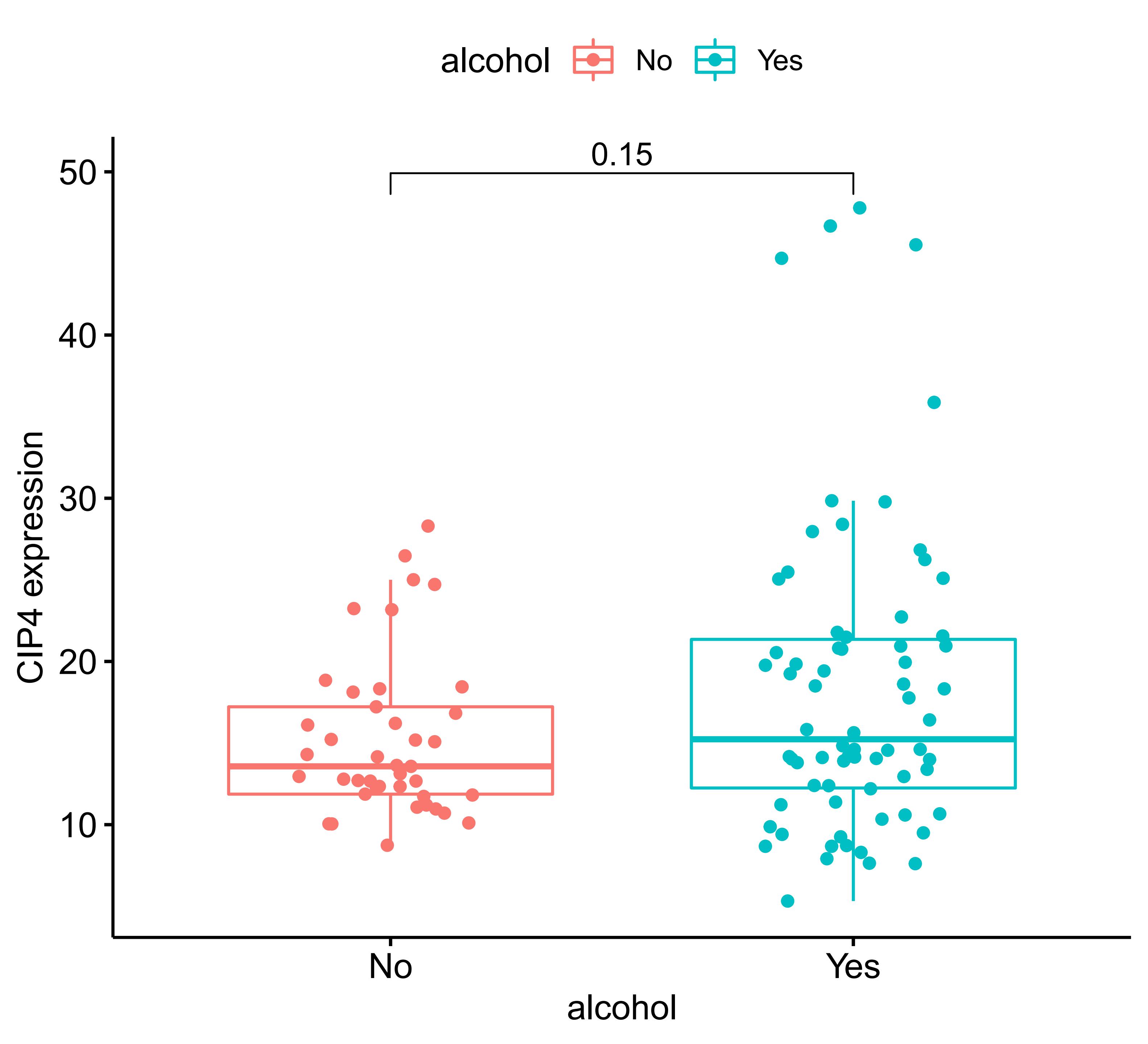

Supplement: S6 Fig — (JPG) [file pone.0253545.s006.jpg]

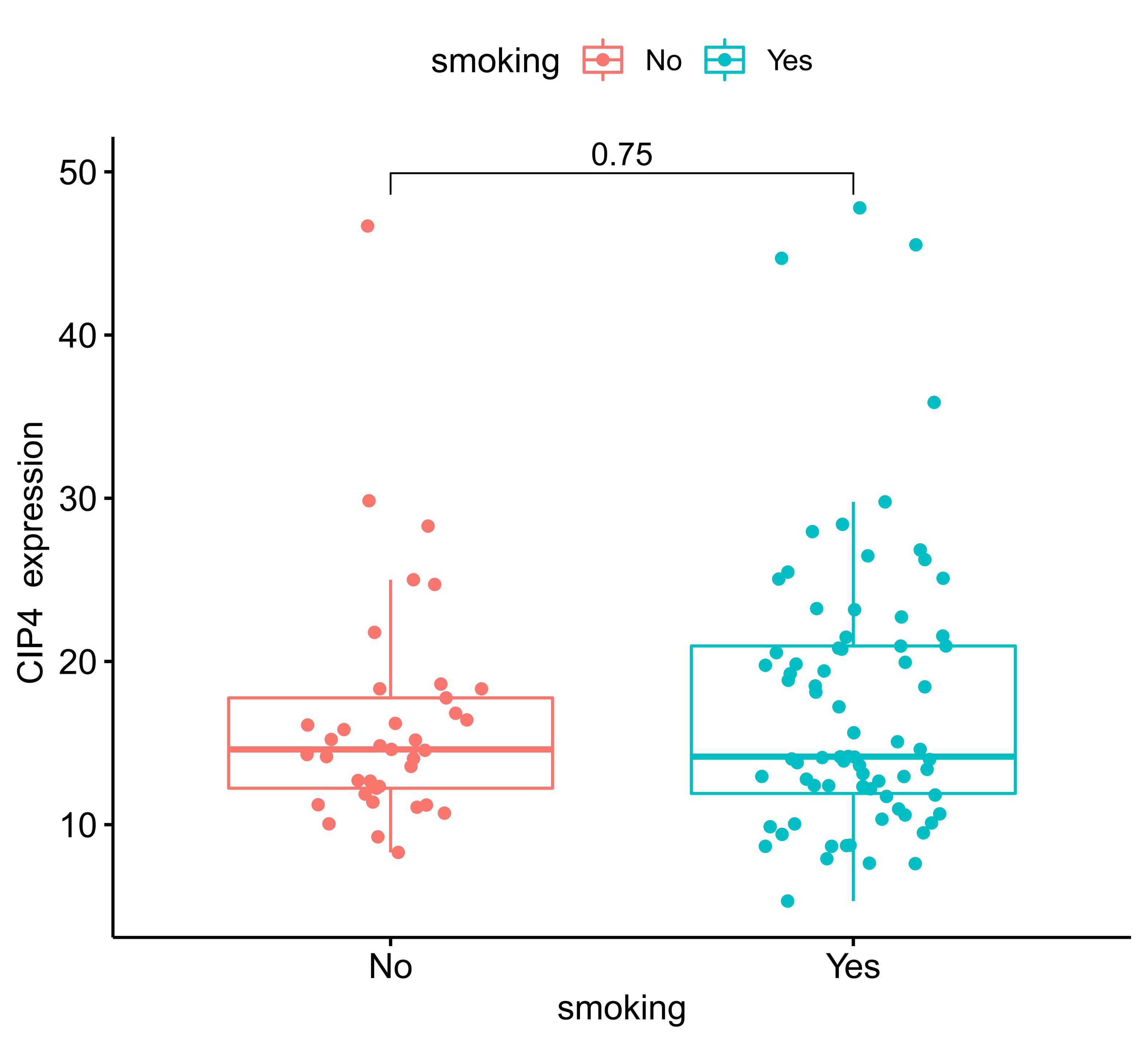

Supplement: S7 Fig — (JPG) [file pone.0253545.s007.jpg]
